# The Role of Ethylene in Plant Responses to K^+^ Deficiency

**DOI:** 10.3389/fpls.2015.01153

**Published:** 2015-12-22

**Authors:** Daniel P. Schachtman

**Affiliations:** Department of Agronomy and Horticulture and Center for Plant Science Innovation, University of Nebraska–LincolnLincoln, NE, USA

**Keywords:** potassium, deficiency, ethylene, reactive oxygen species, roots

## Abstract

Potassium is an essential macronutrient that is involved in regulating turgor, in driving plant growth, and in modulating enzyme activation. The changes in root morphology, root function, as well as cellular and molecular responses to low potassium conditions have been studied in the model plant *Arabidopsis* and in other plant species. In *Arabidopsis* ethylene plays a key role in roots in the transduction of the low potassium signal, which results in altered root function and growth. The first clues regarding the role of ethylene were detected through transcriptional profiling experiments showing changes in the expression of genes related to ethylene biosynthesis. Later it was shown that ethylene plays a foundational early role in the many responses observed in *Arabidopsis*. One of the most striking findings is the link between ethylene and reactive oxygen species (ROS) production, which is part of the signal transduction pathway in K^+^ deprived plants. This mini-review will summarize what is known about the role ethylene plays in response to low potassium in *Arabidopsis* and other plant species.

Ethylene is a plant hormone whose biosynthesis and signal transduction pathways have been well-elucidated (Merchante et al., 2013). The active ethylene substance was discovered as early as the turn of the 20th century and the manipulation of ethylene has been very important in the post-harvest physiology and management of climacteric fruit. Using mutants of *Arabidopsis* that are sensitive and insensitive to the action of the hormone or precursors of ethylene biosynthesis and blockers has led to the discovery of many molecules involved in ethylene perception and action. In *Arabidopsis* the ethylene receptors (*ETR*, *ENS*, and *EIN4*) have been identified along with many of the downstream molecules involved in the progression of the ethylene initiated signal such as *EIN2*, *CTR1*, *ELFs*, *EILs*, and several other molecules (Merchante et al., 2013). One of the most interesting features of studying the role of ethylene in signal transduction pathways is that both insensitive mutants and mutants where the ethylene response is constitutively active are available. In addition to this array of mutants there are also several chemicals that block ethylene responses, such as Ag^+^, 2-aminoethoxyvinyl-glycine (AVG), as well as chemicals such as ethephon that stimulate ethylene production. Due to the excellent biological tools that have been developed in the model plant *Arabidopsis* and the array of different inhibitors, research is well-positioned to study ethylene’s role in signal transduction pathways and to test hypotheses related to the role of ethylene in higher plants in the context of nutritional stresses.

About 10 years ago Shin and Schachtman (2004) embarked on studies to identify the signal transduction cascade that led from the perception of low levels of potassium to the long term adaptive responses that occur, such as changes in shoot growth and inhibition of lateral root growth. The first indication that ethylene was involved in this signal transduction cascade came from a microarray experiment in which whole *Arabidopsis* plants were deprived of potassium for 6 and 30 h followed by a microarray analysis of roots to identify up and down regulated genes (Shin and Schachtman, 2004). Before these experiments in *Arabidopsis* very little was known about the responses to low potassium that occur in plants, except for the well-known phenomenon of the induction of high affinity K^+^ uptake that was described by Epstein (Epstein and Hagen, 1952) and formed the basis for the first studies on high vs. low affinity uptake mechanisms in roots. These early microarray experiments provided new information to generate multiple hypotheses about different hormones, small molecules, and proteins involved in the complex response to low potassium conditions (Shin and Schachtman, 2004). Two clear responses observed in these microarray experiments were the changes in expression of genes related to reactive oxygen species (ROS) production and ethylene (Shin and Schachtman, 2004; **Figure 1**). Two genes related to ethylene biosynthesis [1-aminocyclopropane-1-carboxylic acid (ACC)] were upregulated and one gene related to ethylene perception (*ETR2*; Sakai et al., 1998) was upregulated. From these findings the hypothesis was developed that ethylene production increased under K^+^ deprived conditions. This was tested by placing *Arabidopsis* seedlings into vials containing nutrient solution with and without potassium and analyzing the headspace for ethylene. This biochemical test showed that increased amounts of ethylene were produced upon deprivation of K^+^, validating the hypothesis developed from gene expression data (Shin and Schachtman, 2004).

**FIGURE 1:**
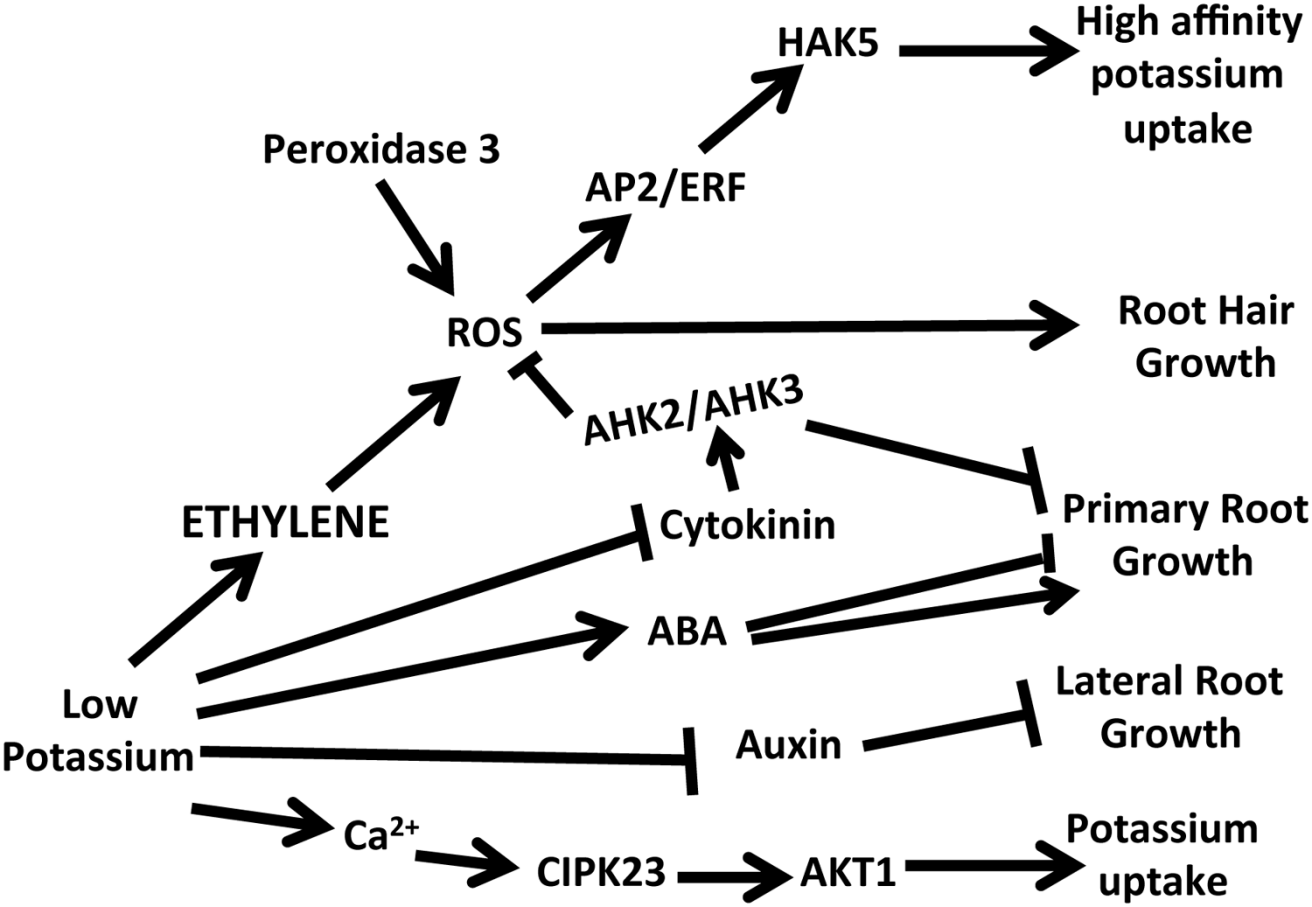
**A schematic of the hormone and calcium responses in *Arabidopsis* following potassium deprivation.** Low potassium leads to increases in ethylene, ABA and calcium and decreases in cytokinin and auxin. Changes in these hormone levels are linked to changes in potassium uptake and different aspects of root growth.

To fully develop an understanding of ethylene’s role in the low potassium signal transduction cascade, studies were initiated using both ethylene mutants and ethylene inhibitors. Experiments using ethylene insensitive mutants (*ETR* and *EIN2*) and a mutant in which the ethylene signaling pathway was constitutively active (*CTR1*) due to the mutation in a negative regulator showed that these lines responded differently from wild type to a low potassium stress (Jung et al., 2009). Key phenotypic changes in these mutants showed that ethylene is important to the changes to root length, shoot fresh weight, and chlorophyll content that occur upon K^+^ deprivation. The changes in these phenotypes that were seen in wild type *Arabidopsis* were not observed in the two different ethylene insensitive mutants and were obvious in the constitutively active mutant in the absence of low K^+^ stress.

Since ROS was previously shown to be involved in the signal transduction pathway in response to low K^+^, experiments were designed to determine whether the ethylene response was up- or down-stream from the induction of ROS. Results showed that when ethephon was added to stimulate ethylene production, the ROS levels in *Arabidopsis* roots increased. Also, the constitutively active ethylene mutants *ctr1* and *eto* all had elevated levels of ROS whereas in several insensitive mutants (*etr*, *ein*) the levels of ROS induced by ethylene were reduced upon K^+^ deprivation (Jung et al., 2009). In the case of the insensitive mutants a slight increase in ROS was detected in the roots, which was much smaller than the increases seen in the wild type. This suggested that in potassium-deprived *Arabidopsis* roots there are also ethylene-independent pathways in response to low potassium (Jung et al., 2009).

Ethylene and ROS are both early in the low K^+^ signaling network. To study how ethylene modulates the downstream signaling responses and to confirm the role of ethylene in the low K^+^ signal transduction cascade, the expression was monitored of a high affinity K^+^ transporter (*HAK5*), whose expression is triggered by low K^+^ conditions. A promoter luciferase construct was used to study the effects of inhibitors and ethephon and real time PCR was used to study *HAK5* expression in several different ethylene mutants (Jung et al., 2009). The analysis of the promoter luciferase constructs showed that ethephon could induce expression of *HAK5* under full nutrient conditions and that inhibitors of ethylene reduced expression but did not totally eliminate expression under low K^+^ conditions (**Figure 1**). This result supports the inference that there is also an ethylene-independent pathway in the low potassium signal transduction pathway. In the mutant of *ein2-1* that is insensitive to ethylene *HAK5* expression was reduced under low K^+^ conditions whereas in two triple mutants (*etr1-6*, *etr2-3*, *ein4-4* and *etr2-3*, *ers2-3*, *ein4-4*) in which ethylene signaling is constitutively active, *HAK5* expression was elevated under full nutrient conditions.

In contrast to the inhibition of lateral root growth that is observed upon deprivation of potassium in *Arabidopsis* (Shin and Schachtman, 2004), root hair growth is increased (Pitts et al., 1998; Jung et al., 2009; **Figure 1**). In K^+^ deprived *Arabidopsis* longer root hairs would be expected because more ethylene is produced in response to low K^+^. In studies on *Arabidopsis* the ethylene insensitive mutants were tested as well as the inhibitors AVG and Ag^+^ (Jung et al., 2009). Results showed that the ethylene insensitive mutants did not alter the elongation of root hairs under low K^+^, but inhibitors even at very low concentrations did abolish the root hair elongation in response to low K^+^. These results on root hair elongation correspond to what was found with the induction of root ROS, where inhibitors, but not the mutants tested completely abolished the increase in ROS. These results support the conclusion that ethylene is a positive regulator of root hair length under low K^+^ conditions.

In addition to the phenotypes described above related to root growth and root hair elongation, the formation of root cortical aerenchyma (RCA) has been highlighted as an important mechanism involved in root adaptation to low concentrations of nutrients such as potassium, nitrogen, and phosphorus (Postma and Lynch, 2011). This single adaptive change in root structure has been shown to increase maize growth under low K^+^ conditions by 72% (Postma and Lynch, 2011). Although there are no data demonstrating a direct role for ethylene under low K^+^ conditions in triggering RCA formation, it has been shown that ethylene is involved in signaling RCA formation (Drew et al., 2000a,b). Together the increase in ethylene under low K^+^ conditions and the strong adaptive advantage of RCA under low K^+^ conditions suggest the two may be linked, and therefore warrant further experimentation to demonstrate this as a possible avenue for increasing plant growth under low K^+^ conditions.

As part of the studies related to signal transduction processes involved in *Arabidopsis* root responses to low K^+^ it was shown that the downregulation of *MYB77* (Shin et al., 2007) plays a role in the reduction of lateral root growth under low potassium conditions. Ethylene may also play a role in reducing lateral root growth in K^+^ deprived roots as it has been shown to decrease lateral root growth in *Arabidopsis* (Lewis et al., 2011). In roots of plants grown in full nutrients the response to ethylene is mediated by high rates of auxin transport via *PIN3* and *PIN7*, which prevents the localization of auxin that is needed for lateral root formation (Lewis et al., 2011). Under low K^+^ conditions there may be important interactions between ethylene and auxin that lead to altered lateral root growth.

Similar to what has been shown in *Arabidopsis*, several genes involved in ethylene biosynthesis and signaling were upregulated by K^+^ deprivation in watermelon (*Citrullus lanatus* Thunb.; Fan et al., 2014). In that study two varieties of watermelon with contrasting K^+^ efficiencies were used for transcriptional profiling to identify changes in gene expression and to reveal differences in the molecular basis of varietal differences. The roots of the watermelon varieties were profiled 6 and 120 h after K^+^ deprivation. At the 120 h time point ACC was upregulated, as well as genes related to ethylene perception and signaling including: *ERF1*, *ERF-1*, and *EBF1* in the less efficient watermelon variety (8424; Fan et al., 2014). At the earlier stage, 6 h after deprivation, several ethylene-related genes were downregulated highlighting a slight difference between *Arabidopsis* and watermelon. In another study that compared barley lines that differed in response to low potassium the genes involved in ethylene biosynthesis or signaling were either not altered by low potassium or down regulated (Zeng et al., 2014). These gene expression differences across plant species could be due to different signaling pathways, or more likely differences in physiological processes involved in response time to low K^+^.

Aside from studies on *Arabidopsis* and watermelon roots there is one other study that characterized the ethylene response of sunflower leaves in plants that were grown under potassium deficient conditions. In sunflower an interesting interaction was shown. Closure of stomata due to drought was partially short circuited by K^+^ deprivation. Stomatal conductance of droughted and K^+^ deprived plants was higher than plants that were only drought stressed (Benlloch-Gonzalez et al., 2008). In this system the leaves of the drought stressed and K^+^ deprived sunflower plants were shown to be producing more ethylene, which was correlated with increased stomatal conductance (Benlloch-Gonzalez et al., 2010). To further strengthen the correlation between ethylene and stomatal conductance under low K^+^ conditions, cobalt was used as an inhibitor of ethylene synthesis. When ethylene was inhibited by low levels of cobalt, plants showed a decrease in stomatal conductance under low K^+^ conditions lower than wild type. Under low K^+^ conditions in sunflower ethylene plays a role in modulating stomatal conductance under drought, which may be an adaptive response that increases the transport of K^+^ from roots to leaves (Benlloch-Gonzalez et al., 2010).

Ethylene plays very important roles in the *Arabidopsis* signal transduction network for plant adaptation to low potassium conditions as well as deprivation of other nutrients including phosphorus, sulfur, iron and nitrogen (Garcia et al., 2015). In *Arabidopsis* ethylene is an early signaling molecule that leads to the induction of ROS and ultimately to the expression of a high affinity potassium transporter that is important in the uptake of K^+^. Ethylene also plays a role in root hair elongation and in modulating stomatal conductance in response to drought and low K^+^. Although these findings suggest a key role for ethylene in plant response to low K^+^ there are still many open questions. One major question that has not yet been explored is the cross talk among ethylene and the other hormones (**Figure 1**) that have been shown to be important under low K^+^ conditions (Armengaud et al., 2004; Schachtman and Shin, 2007; Shin et al., 2007; Kim et al., 2009; Nam et al., 2012) as well as the interactions with other nutrient stresses (Iqbal et al., 2013).

## Author Contributions

DS wrote this review.

## Conflict of Interest Statement

The author declares that the research was conducted in the absence of any commercial or financial relationships that could be construed as a potential conflict of interest.
